# Towards the Autonomy: Control Systems for the Ship in Confined and Open Waters

**DOI:** 10.3390/s21072286

**Published:** 2021-03-24

**Authors:** Anna Miller, Monika Rybczak, Andrzej Rak

**Affiliations:** Department of Ship Automation, Gdynia Maritime University, 81-85 Morska Str., 81-225 Gdynia, Poland; a.miller@we.umg.edu.pl (A.M.); a.rak@we.umg.edu.pl (A.R.)

**Keywords:** marine autonomous surface ship, MASS, ship motion control, control data visualization, scale ship model

## Abstract

The concept of the Marine Autonomous Surface Ship (MASS) requires new solutions in many areas: from law, through economics, social sciences, environmental issues to the technology and even ethics. It also plays a central role in the work of numerous research teams dealing with the ship motion control systems. This article presents the results of the experiments with application of the selected control methods in automatic steering of the movement of an autonomous ship in the two regimes: during the maneuvers at low speed (in a harbor confined waters) and during the lake trials in open water conditions. In the first case, multidimensional state controller synthesized with Linear Matrix Inequalities (LMI) algorithms was used, while, in the second case, Model Predictive Control (MPC) control was adopted. The object for which the experiments were carried out was 1:24 scale model of the Liquefied Natural Gas (LNG) carrier. The paper presents also the design of the measurement and control system and the user interface. The experiments were conducted in the natural conditions on the lake. The results of the experiments indicate the fundamental role of the measurement system in the process of controlling an autonomous ship.

## 1. Introduction

Contemporary scientific research related to the marine control field is carried out i.a. as part of the “Autonomous Ships” project in Norwegian University of Science and Technology, “Autoship” project in Horizon2020, “The Mayflower Autonomous Ship” concept by the Promoting Marine Research and Exploration (PROMARE) and Autonomous Vessel with an Air Look (AVAL) project conducted in Poland. The number of research activities carried out and the interest of scientists point to large application possibilities of the created solutions. All these projects are interdisciplinary ones involving cooperation of the hydrodynamicians, electricians, navigators, and control professionals.

According to the researchers’ experience in the marine control field, Marine Autonomous Surface Ship (MASS) ship control systems can be divided into four main areas.

- The first area is an autonomous calculation of the optimal trajectory also known as ships autonomous navigation [1,2,3,4,5]. Generating automatic trajectories for navigational maps, that include harbor infrastructure, like piers, was partially described in References [6,7,8]. In Reference [9], a novel three-step approach for WSL (Water-Shore Line) detection is, therefore, proposed to solve this problem through the information of an image sequence. Firstly, the initial line segment pool is built by the line segment detector (LSD) algorithm.
- Verification of the proposed control algorithms should take into consideration safety at sea rules, like in Reference [10]. In Reference [11], analysis of the autonomous ship is explored, and system-theoretic process analysis (STPA) and the functional resonance analysis method (FRAM) are identified as the most representative new methods that can be used for hazard analysis of autonomous ships.
- In the third area, autonomous ship control is connected with power management [12,13]. In Reference [14], the authors show the importance of autonomous power management, its impact on fuel consumption, and the need to use intelligent, self-learning algorithms.
- The four area is a concept of autonomous ship control for both cruising and maneuvering speeds. For example, one can refer to the project called Advanced Autonomous Waterborne Applications Initiative (AAWA) created by Rolls-Royce and Kongsberg, described in References [15,16]. Project of the autonomous transport system applicable for the coastal waters and areas beyond the inlands is described also in References [17,18].

However, the above four areas should be integrated already from design stage all the way through calculations, navigation and safety to be a real assistance for the ship’s operators. This kind of control was described in 2018 by the International Maritime Organization (IMO) and called MASS (Maritime Autonomous Surface Ships) [19]. This IMO standardization has defined the following four levels of operation for MASS. The first level is a manned ship with automated processes and decision support. The second, a remotely controlled ship with seafarers on-board. Third is a remotely controlled ship without seafarers on-board. The fourth level it is a fully autonomous ship [20]. As described in Reference [21], augmentation of present IMO-mandated vessel environmental sensor systems with future capability is essential to achieving situational awareness for MASS and ensuring proper supervision and traceability of decision-making. Questions are now being asked whether smart ships should be fully autonomous, remote controlled, or manned with a skeleton crew, and who would ultimately be responsible for the ship in question and how smart ships would affect sea traffic. Some of these problems have been discussed in Reference [22]. With great precision and direct indication of many sources describing MASS issues, as well as problems that are posed in science, are presented in Reference [23].

As described in References [24,25], autonomous navigation decision-making system is the core of a MASS technology, and its effectiveness directly determines the safety and reliability of navigation, playing a role similar to a human *’mind’*. During a voyage, the thinking and decision-making process is very complex. This article will present the results of the research carried out on Silm Lake in Iława, Poland, for the “Dorchester Lady” training ship model. Additionally, visualizations of the MASS processes are presented.

Usually, ship motion control research is tested on the software simulation models. The main reason for such approach is cost of full scale ship usage. The model of the Liquefied Natural Gas (LNG) tanker used in this project to test the control system is built in large scale (1:24), which gives very good approximation of conditions for real sea-going ship. The model is fitted partly with real marine navigation equipment, too. This factor gives uniqueness to this research.

In MASS, there is a need of reference safe trajectory generation and control subsystems cooperation. The first of them is usually an anti-collision system [2], the result of which is a set of waypoints (WPTs) defining safe ship trajectory. These WPTs are computed according to the International Regulations for Preventing Collisions at Sea (COLREG). Control subsystem is designed to provide the ability to move along the designated route. As it was mentioned in Reference [23], there are 4 types of control in MASS: speed control, course control, stabilization control, path-following and trajectory tracking. Speed control, separately, does not really find application in MASS because it is based on following of route. It may be a part of trajectory tracking system, combined with course control, which will by discussed in a detailed way in Section 3.1 of this publication. Stabilization control in MASS is applied for service vessels, where dynamic positioning is a main task of the control system, i.e., PID control with feedforward action [26], robust adaptive control [27], or state-space control [28]. Path-following and trajectory tracking are the most commonly used strategies in MASS. Path-following, in contrast to the trajectory tracking, does not require ship to be at a certain WPT at a certain time. So, it is the most common concept in the MASS automatic control. One can apply relatively simple methods as PID control scheme with switching approach for different operating conditions [29], more computationally complicated ones as optimal robust control combined with roll stabilization [30], and also use artificial intelligence, applying neural path following controller to the ship [31]. Trajectory tracking is also popular in MASS control concept, i.e., using sliding mode control [32], robust adaptive control [33], or artificial intelligence [34]. The overall concepts of the research trends in path-following and trajectory tracking are convergent. Beyond them, predictive techniques, also proposed in Section 3.2, have their place [35,36], as well.

## 2. Training Ship

**Figure 1:**
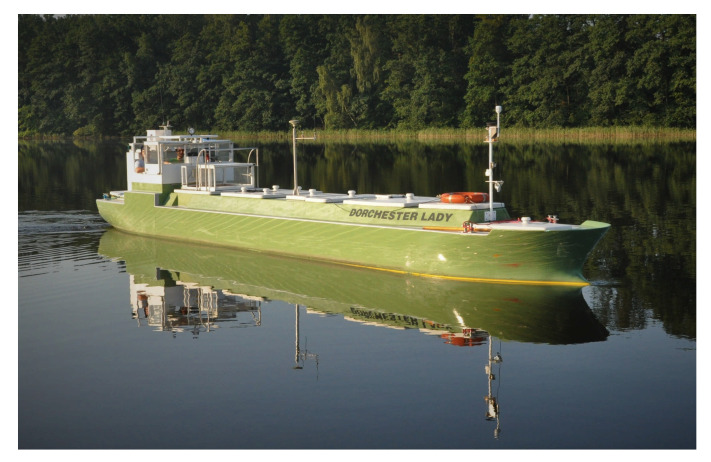
“Dorchester Lady” training ship—scale model of 113,500 Deadweight Tonnage (DWT) Liquefied Natural Gas (LNG) carrier.

Conducting research on an autonomous ship requires: safe trajectory generation system, appropriate controller, and vessel equipment adapted for automatic control. Today, due to the lack of relevant regulations, it is not possible to use real commercial vessels for scientific research. Therefore, a scaled-down floating training ship, one of the small fleet owned by Foundation for Safety of Navigation and Environment Protection, was used. This ship, Liquefied Natural Gas (LNG) Carrier “Dorchester Lady”, presented in Figure 1, was adapted for autonomous shipping. She is described in detail in References [37,38,39].

The training ship has been built in scale 1:24 according to geometric, kinematic and dynamic similarity laws. Only the Reynolds number cannot be kept constant, due to the fact that ship and model move in the same environment; so, the complete kinematic and dynamic similarity to the full scale ship is not obtained. This leads to relevant seagoing ship dynamics mapping for training and research purpose. Training ship particulars are presented in Table 1.

The model of LNG carrier is equipped with two DC motor driven azipods with counter-flow propellers, a tunnel thruster and azimuth thruster, both located on the bow. The model operates in manned mode using signals from the gyrocompass and Global Positioning System (GPS) receiver. External disturbances, like wind force and direction, are measured by the anemometer. Training ship positions are determined with centimeter accuracy do to GPS system working in the Real Time Kinematic (RTK) mode. Measurable external disturbances, like wind force and direction, may be used as inputs in the optional feedforward controller.

## 3. Automation of the Ship Motion Control Processes

Automation of the ship motion control process requires synthesis of the control system for the desired vessel. It is based on the control law creation and its application to a real vessel. In general, three types of control modes are distinguished: trajectory tracking, path following, and reference speed tracking.

**Figure 2:**
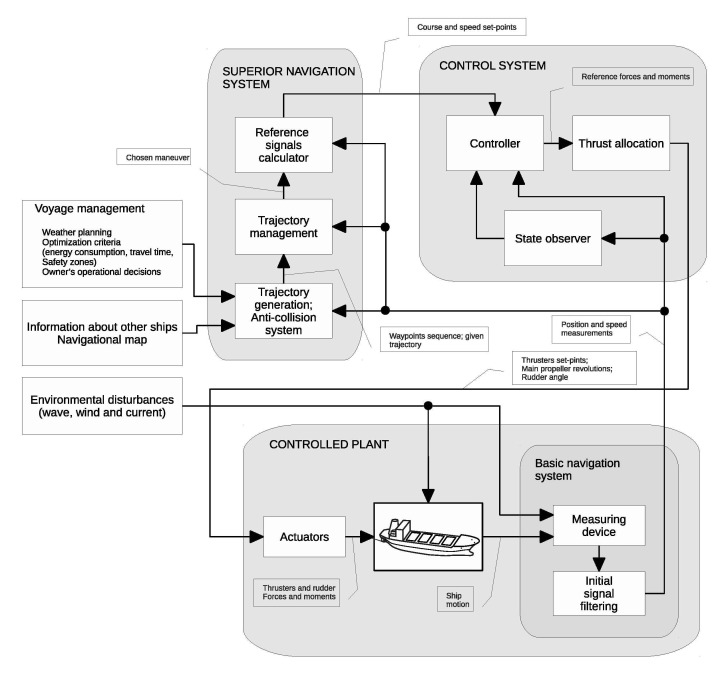
General arrangement of the automatic ship motion control system.

Fully functional MASS requires three subsystems cooperation (Figure 2):

– **Supervisory navigation system**—where the safe trajectory is generated based on the waypoints sequence, voyage management data, and information about other ships moving in the vicinity, taking into account International Regulations for Preventing Collisions at Sea (COLREG).
– **Control system**—where, based on the course and speed reference signals, desired actuators’ commands are computed. In this subsystem, the controller cooperates with the state observer and thrust allocation system for low speed multidimensional control.
– **Controlled plant**—ship equipped with controllable actuators and measurement devices.

Firstly, an algorithm based on Linear Matrix Inequalities (LMI) was tested for low speeds, but it did not work in open water. Hence, research was started to develop another control algorithm for high speed, and, in this case, Model Predictive Control (MPC) was the right choice. The “failed” results are not presented here due to limitation of the tex length.

The advantage of the controller based on linear matrix inequalities is the size of the gain matrix K = [3 × 6]; additionally, the multidimensional control for low speeds worked perfectly during the verification on the lake, where, as the figure shows for individual speeds *u*, *v*, *r*, there was no cross-coupling, which is a great advantage of this research.

MPC is a control strategy using an internal ship’s model in order to predict her future motion. The ship is characterized by high inertia, which makes the control more complicated and reduces its quality. MPC usage allows inclusion of the ship dynamics into future control signals calculation process. So, due to these advantages, this control scheme is applicable for such plants as ships, and our research experience shows that it may be successfully applied to the MASS.

### 3.1. Multidimensional Control of Autonomous Ship Maneuvering in Port

**Figure 3:**
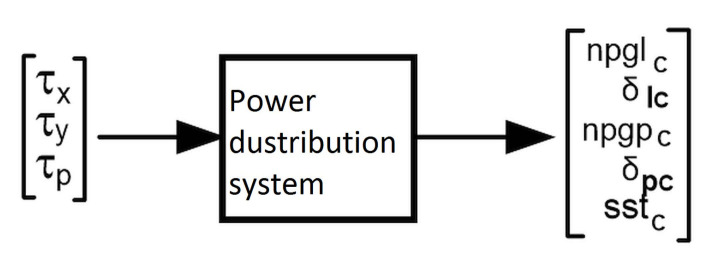
Distribution of the three control signals to the drive device commands: $npgl_{c}$, ($npgr_{c}$)—rpm’s of the left (right) azipod propeller, $\delta_{l}c$, ($\delta_{r}c$)—left (right) pod rotation angle, and $sst_{c}$—relative force of the bow tunnel thruster.

Ship control algorithm for movement on restricted area along a selected trajectory was created using linear matrix inequalities (LMI). As a restricted area, we mean confined waters, like harbor area or lock entrance. The control object, the “Dorchester Lady”, ship model is a nonlinear object, especially at low velocities, since, during ship dynamics, modeling for controller synthesis linearization of the model around its working point was used. The identification process of a model took into consideration:

- stationary Kalman filter system [40] (this system is used for u,v,r velocities estimation), because “Dorchester Lady” ship model was not equipped with instruments for measuring linear velocities and thus, the need exists for Kalman filter system,
- thrust allocation system used for calculating three components of vector:

(1) $$u={\left[\tau_{x},\tau_{y},\tau_{r}\right]}^{T},$$

to vector *T* with seven components of propulsion devices control signals. Controlled object has three input signals: $\tau_{x},\tau_{y},\tau_{p}$, and three output signals: $\hat{u},\hat{v},\hat{r}$, where: [$\tau_{x}$] is the reference force (thrust) on the ships longitudinal axis, [$\tau_{y}$] is the reference force (thrust) on the ships lateral axis, and [$\tau_{r}$] is the reference rotational moment. The method of power distribution between the individual propellers is determined by the number and type of devices installed and their arrangement in or under the hull. Therefore, there is no single commonly used algorithm, and each such arrangement is basically designed individually. In the case of the training vessel, this system is based on Moore-Penrose pseudo-inverse matrix calculations. The method used in the allocation was analogous to those described for the thrust allocation of the “Blue Lady” model ship in References [38,41]. Figure 3 shows the signals transmitted from the controller to the allocation systems.

**Figure 4:**
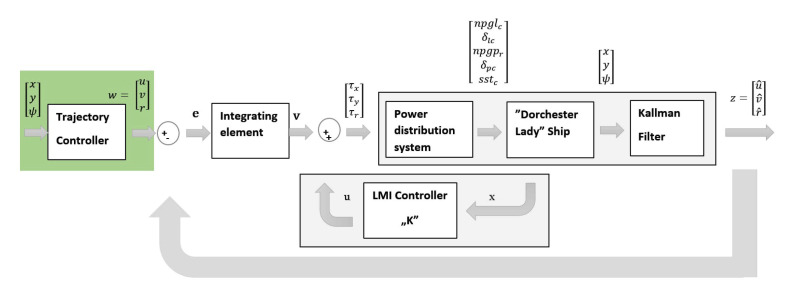
Block diagram system autonomous with trajectory controller (green area) and velocities controller (gray area).

The diagram in Figure 4 shows that the autonomous ship steering in the low speed consists of two main stages. The first stage concerns the synthesis of the low speed regulator based on the LMI. The second stage, marked in green on the same diagram, concerns the synthesis of the trajectory regulator. This means that controller input signals are differences between reference and filtered velocity signals. And controller output signals are three force signals, $\tau_{x}$, $\tau_{y}$, and $\tau_{p}$, which are sent to the thrust allocation system.

Matrices **A**, **B**, and **C** of the controlled object, the “Dorchester Lady” ship model, have the below form:

(2) $$\begin{array}{c} A=\left[\begin{array}{ccc} -1.00\cdot 10^{-2} & 0 & -1.20\cdot 10^{-2}\\ -4.70\cdot 10^{-3} & -2.48\cdot 10^{-2} & -1.73\cdot 10^{-2}\\ -9.30\cdot 10^{-3} & 0 & -3.11\cdot 10^{-2} \end{array}\right]\\ B=\left[\begin{array}{ccc} 3.83\cdot 10^{-5} & 0 & -1.17\cdot 10^{-7}\\ 9.08\cdot 10^{-8} & 1.42\cdot 10^{-5} & 1.97\cdot 10^{-7}\\ -3.51\cdot 10^{-6} & 0 & 5.35\cdot 10^{-5} \end{array}\right]\\ C=\left[\begin{array}{ccc} 1 & 0 & 0\\ 0 & 1 & 0\\ 0 & 0 & 1 \end{array}\right]\;D=\left[\begin{array}{ccc} 0 & 0 & 0\\ 0 & 0 & 0\\ 0 & 0 & 0 \end{array}\right]. \end{array}$$

Basic canonical form of linear matrix inequalities is (based on Reference [42]):

(3) $$\;F\left(x\right)=F_{0}+\sum_{i=1}^{m}F_{i}x_{i}≻0,\;$$

where:

- decision variable vector (unknown) *x*, ${\left[x_{1},x_{2},...,x_{m}\right]}^{T}$$\in R^{m}$,
- matrices marked as $F_{0}..Fi\in R^{nxn}$ are real and symmetrical, where symmetrical matrix has the form of: $F_{i}=F_{i}^{T}$ for i=0,...,m, and
- the term “≻0” means that the matrix F(x) is positively defined.

**Figure 5:**
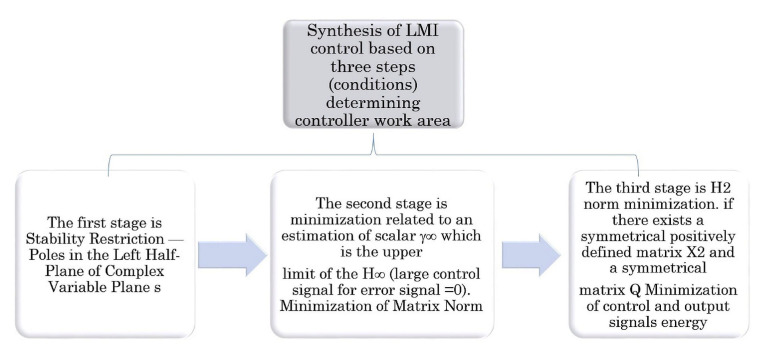
First stage of autonomous ship control. Synthesis of the low speed regulator.

LMI conditions create a convex set of limitations that has to be formulated for state space controller synthesis process. The synthesis of the regulator is based on three conditions shown in Figure 5.

This means calculating gain matrix K for the state space controller described by the formula:

(4) Y=−K·X.

To calculate matrix **K**, one must know the values of matrices **X** and **Y**, which are calculated using an optimization software based on defined LMI conditions. For this we assume that matrix **X** is symmetrical and positively defined and that it’s inverse, real matrix **Y**${}^{-1}$ exist. “Yalmip” and “SeDuMi” libraries for MATLAB software were used for controller synthesis [41,43]. After the calculations, the controller matrix **K** has the below form:

(5) $$K=\left[\begin{array}{cccccc} 1595.2 & -0.00 & 0.10 & -807.8 & -0.00 & 0.00\\ -0.01 & 1664.8 & -36.00 & 0.00 & -897.90 & 2.00\\ -6.10 & -28.00 & 435.40 & 35.00 & 16.00 & -23.44 \end{array}\right].$$

The important fact is that control matrix is a full matrix (and not diagonal), which means that all three velocities are controlled at the same time and are interconnected.

### 3.2. Autonomous Ship Open Water Trajectory Tracking

**Figure 6:**
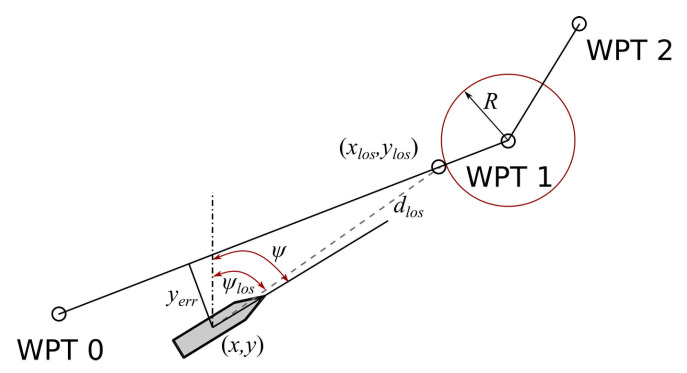
Ship’s position fixing in relation to the reference trajectory.

Autonomous ship moving at operational speed is a problem classified as open water ship motion control. One of the methods for determining the trajectory return path, a straight line along which ship is returning to the reference trajectory, is based on the ships return course computation. Line-of-sight (LOS) algorithm may then be used [44]. It is determined based on three consecutive waypoints: passed, closest, and next one, combined with the present ship’s position. The example of the ship’s positioning relative to the reference trajectory is shown in Figure 6.

Intersection of the return course ($\psi_{los}$) with the reference trajectory ($x_{los},y_{los}$) is determined by the cross–track error ($y_{err}$) and line length ($d_{los}$). Trajectory return course is defined by the equation [44]:

(6) $$\psi_{los}=atan2\left(y_{los}-y,\;x_{los}-x\right),$$

where (x,y) is a current ship’s position.

Trajectory tracking controller at operational speed, for the "Dorchester Lady" training ship, was created with the use of Model Predictive Control (MPC) technology. Internal plant model was identified with the use of MATLAB System Identification Toolbox. Due to the faster calculation time, it was decided to use linearized state space model describing the relationship between azipod angle of rotation (δ) and ship’s rotational velocity (*r*). The predictive incremental state-space model [38] has the form described below:

(7) $$\begin{array}{cc} x_{k+1} & =A\cdot x_{k}+B\cdot u_{k}+K\cdot e_{k} \end{array}$$

(8) $$\begin{array}{cc} y_{k} & =C\cdot x_{k}+e_{k}, \end{array}$$

where:

- $x_{k+1}$—predicted next state;
- $x_{k}$—predicted current state;
- $u_{k}$—current control signal (azipod angle of rotation δ);
- $y_{k}$—current output signal (rotational velocity *r*).

Matrices **A**, **B**, **C**, and **K** of the controlled object, the “Dorchester Lady” ship model, have the form:

(9) $$\begin{array}{ccc} A & = & \left[\begin{array}{cccc} 0 & 1 & 0 & 0\\ 0 & 0 & 1 & 0\\ 0 & 0 & 0 & 1\\ -0.3145 & 1.736 & -3.486 & 3.062 \end{array}\right], \end{array}$$

(10) $$\begin{array}{ccc} B & = & {\left[\begin{array}{cccc} -3.63\cdot 10^{-6} & -2.59\cdot 10^{-6} & 1.75\cdot 10^{-4} & 2.89\cdot 10^{-4} \end{array}\right]}^{T}, \end{array}$$

(11) $$\begin{array}{ccc} C & = & \left[\begin{array}{cccc} 1 & 0 & 0 & 0 \end{array}\right], \end{array}$$

(12) $$\begin{array}{ccc} K & = & {\left[\begin{array}{cccc} 2.142 & 3.558 & 5.034 & 6.436 \end{array}\right]}^{T}. \end{array}$$

The internal identified model links azipod angle of rotation with the rotational velocity in order to allow for future control signal (azipod angle of rotation) predictions.

Sub-optimal control signals are computed during constrained quadratic programming optimization procedure based on the cost function:

(13) $$J=\gamma_{y}\sum_{p=N_{1}}^{N}{\left[r\left(k+p|k\right)-y\left(k+p|k\right)\right]}^{2}+\gamma_{u}\sum_{p=0}^{Nu-1}{\left[\delta u\left(k+p|k\right)\right]}^{2},$$

where:

- $\gamma_{u}$,$\gamma_{y}$—output signal change and error weight coefficients;
- r,y,δu—reference, output, and control signal change values;
- (k+p|k)—signal value at k+p time moment predicted in *k* time moment;
- $N,N_{u}$—prediction and control horizon lengths.

The following numerical values of parameters were adopted: N = 10 [s], $N_{u}$ = 2 [s], $\gamma_{y}$ = 100 [-], $\gamma_{u}$ = 0.5 [-]. Prediction of future output signal values was made using a Kalman filter.

Indeed, the proposed solutions relate specifically to the Dorchester Lady model; we did not present more general identifications. Our knowledge was based on the non-linear training ships model proposed by Reference [37]. The LMI controller applied for the presented MASS was described in a detailed way in References [41,43]. The MPC controller was based on the linaerized incremental model presented in Reference [38].

## 4. Essential Components Arrangement of the Autonomous Training Ship

**Figure 7:**
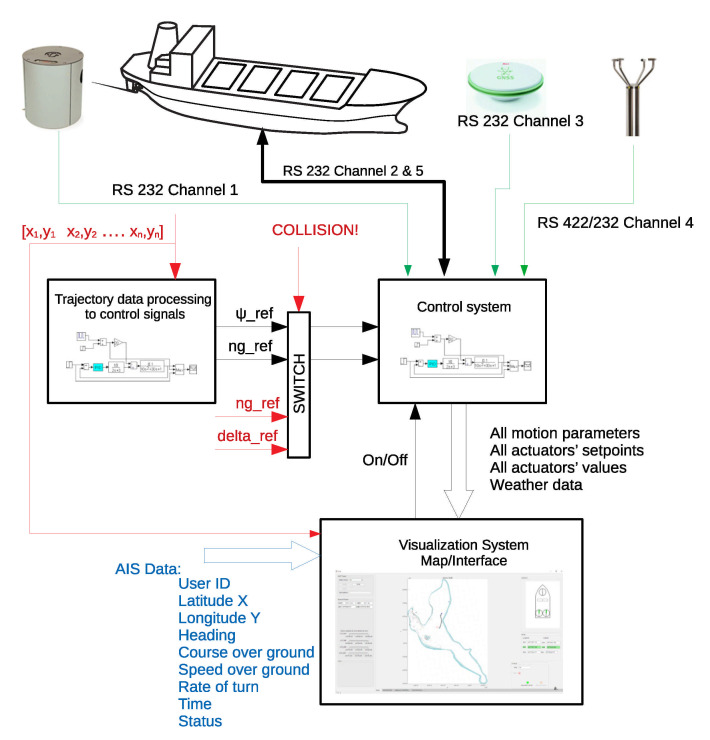
Autonomous training ship motion control system—block diagram of main components.

Training ships used in Iława Ship Handling Research and Training Center are fully functional models of seagoing vessels, used for marine officers training and for research. The “Dorchester Lady” is equipped, i.e., with Anschütz Standard 20 Gyro Compass, GPS Reciver Leica System 1200, and Gill WindObserver ultrasonic anemometer. Signals from the aforementioned navigational equipment are transmitted using National Marine Electronics Association (NMEA) 0183 standard, working on the basis of serial links. Three RS-232 and RS-422 channels are used to connect devices with automatic control system, which is presented in Figure 7. Communication between control system and ship actuators is also realized using RS-232 standard.

Full-mission automatic control system is operating in one of three modes:

– **trajectory tracking**—in which it cooperates with safe trajectory generation subsystem. After defining a safe and achievable trajectory, reference waypoints are transformed into reference control signals—reference course and main engine set-points.
– **maneuvering mode in a restricted area**—where ship movement is defined by the set of waypoints and desired ship’s heading. This is the way the ship moves, e.g., when approaching the quay. Surge, sway, and yaw are controlled. Operation in this mode requires not only azipods usage; bow and azimuth thrusters are also activated to perform necessary motions.
– **Last Minute Maneuver (LMM) for collision avoidance**—where safe trajectory generation subsystem defines thrusters’ setpoints allowing for collision avoidance or minimizing its effects (switch input signals marked by red in Figure 7).

When tracking trajectories at operating speed only main propulsion and a steering gear of the ship are active. LNG carrier “Dorchester Lady” is propelled and steered by the azipods. Maneuvering in a restricted area requires the use of all installed thrusters, which efficiency is high at the low speeds. All thrusters are electrically powered, so it is hardly possible to use them all together working with maximum power. There is a need to use thrust allocation system, which distributes energy between individual actuators based on reference longitudinal and transversal thrusts and rotational moment.

The control system implemented on board of the training ship is designed with the use of MATLAB/Simulink software. Industrial computer IPC934-230-FL equipped with 8-port Quatech Serial Device Server is used as a Simulink Real-Time Target (SLRT)-programmable controller. Application of the hardware solution described above allows for ship’s real-time control, fast prototyping using host computer equipped with MATLAB software and real-time data acquisition for the visualization purposes.

### HMI for Research and Documentation Purpose

Standard navigational data visualization form is to show them on the standard Electronic Chart Display (ECDIS) screen. But, in the presented case, there is a need to control not only reference trajectory, current ship’s position, and heading for automatic control or autonomous operation of the ship. The supervisor during the system tests needs, moreover, knowledge about actuators settings, control errors, control signals’ histories, external disturbances, and operating mode, as well as to have ability to switch to the manual control in case of emergency.

**Figure 8:**
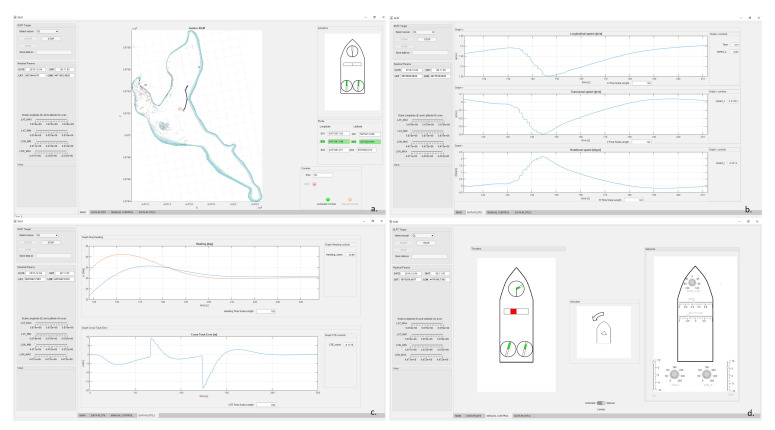
Screenshots of the autonomous training ship control system user interface (Autonomous Training Ship (ATS) Graphical User Interface). (**a**) GUI main screen with map. (**b**) Time histories of ship velocities. (**c**) Time histories of heading and cross-track error. (**d**) Thrusters’ activities display and manual set-point knobs.

Autonomous Training Ship (ATS) data visualization is implemented in a dedicated application built in MATLAB AppDesigner tool. A graphical user interface has been created, for which the main screen shown in Figure 8a has the following functionalities:

– **Autonomous ship selection option**: after choosing a ship to control, in panel “Actuators” thrusters configuration corresponding to those actually installed on the particular training ship is presented. Their setpoints are updated and visualized every second.
– **“Start”, “Stop”, and “Save” buttons**: they are available depending on the state of the SLRT controller software and allow the user to start and stop application and save data from its memory.
– **“Nautical Params” panel**: date, time, and position from GPS receiver are displayed together with sliders allowing for chart scaling.
– **“Route” panel**: data of three consecutive waypoints are displayed there—previous, current (highlighted in green), and next.
– **“Controls” panel**: there are grouped controls indicating ship’s operating mode (automatic or manual control) and whether LMM maneuver is realized, while the name of selected ship is written in the text box.
– **Electronic chart**: where waypoints are presented together with measured ship’s trajectory.

One of the important reasons why the entire user interface has been object-programmed using MATLAB language is the possibility of reading data directly from the controller in real-time and plotting them on the graphs. Exemplary screens demonstrate longitudinal, transversal, and rotational ship’s speeds, heading, and cross track error (Figure 8b,c). User can manually define timescale for each graph separately.

There is also tab called “Manual Control” in the application (Figure 8d). Controls placed there allow for ship’s manual control via SLRT. After switching from “Automatic Control” to “Manual”, all controls located on the right side of screen are enabled. There is possibility to change all thrusters’ setpoints. Their current values are presented on the left side of screen. In the “Velocities” panel, the ship motion vector is shown.

Adjustment of the control signal values may be done via SLRT structure, where all tunable parameters were compiled. If there is any discrepancy between value set in the Target controller and one stored in the SLRT structure, the parameter in the controller is then adjusted. The lack of an efficient data readout mechanism from SLRT Target brought timer usage on. In each time step, all readable parameters from SLRT structure are compared with these displayed in the user interface in previous time step. If there are any differences, displayed values are adjusted.

## 5. Results

In the MASS ship, motion may be divided into two main parts, namely port maneuvers and trajectory tracking. In order to present the way of whole system operation, we have prepared two sample sets of maneuvers. The first of them presents quay departure in confined waters, and the second one presents trajectory tracking in open waters results. The main idea of the first maneuver is to show that there is a possibility to realize safe quay departure in a fully autonomous ship control system. Reference trajectory and course are given by the superior reference trajectory generation subsystem, which takes into account quay, port infrastructure, and fairway signs positions. The role of ship motion system is then restricted to the reference tracking. The LNG carrier is highly non-linear, multidimensional control object. During low-speed, port maneuvers azipods and bow thruster are used, so there is a need of multidimensional control system application. Ship motion in the harbor should be controlled precisely in order not to collide with the other ships and infrastructure in the restricted waters. This approach requires position and course into reference velocities (*u*, *v* and *r*) recalculation. Control quality is assessed then based on them. In the system, it was required that the overshoot for each speed should not exceed 20%. The second described case concerns trajectory tracking under normal operating conditions. We decided to present results of the reference tracking, which is generated by the superior safe trajectory generation system as a set of WPTs. The course is not given in this case by the superior system; it is counted as a bearing between two consecutive WPTs. This system takes into account water depth and changes reference in order not to collide with the other ships. Measure of the quality of regulation is defined as steady-state cross-track error, which cannot exceed ship’s breadth.

The trials of the control systems were carried out in the Ship Handling Research and Training Center in Iława. The multidimensional low-velocities system with the LMI controller was tested in the port area, while the full speed MPC control system in the open waters of the lake.

**Figure 9:**
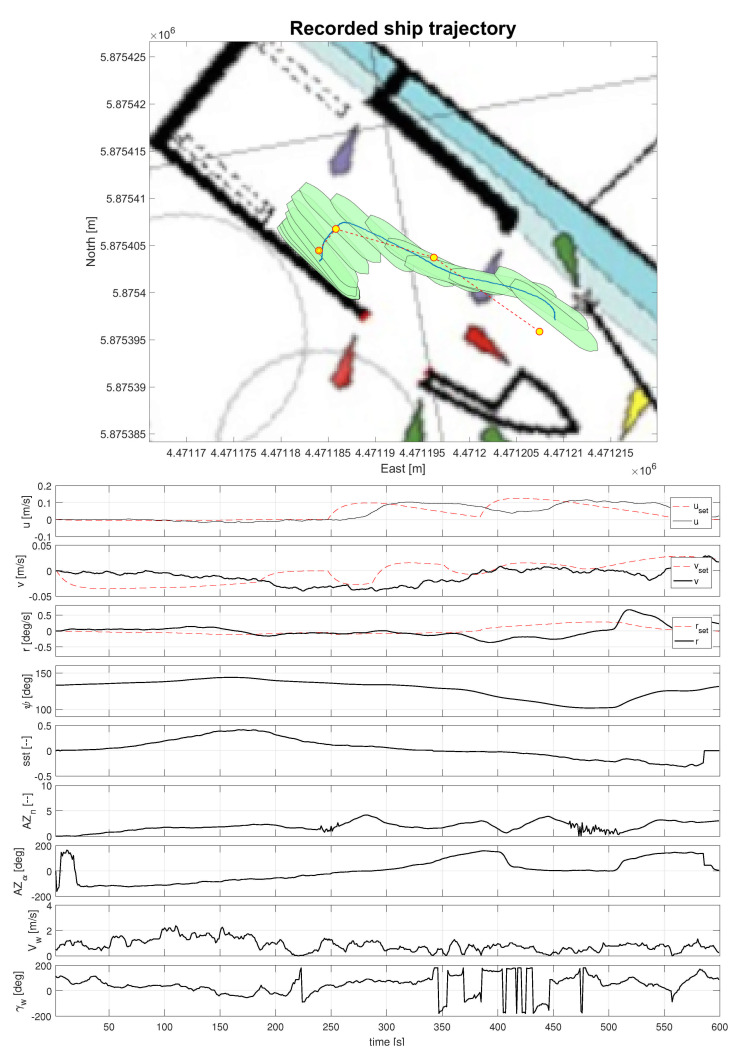
Departure maneuver of the “Dorchester Lady” model from the Iława Port. Upper part: Reference (dashed line) and measured (solid line) trajectories of the training ship. Lower part: recorded signals marked with following symbols: y,v,r—velocities, longitudinal, transversal (“–” sign indicates ship motion to the port side), and rotational (“–” sign indicates counterclockwise rotation), respectively; ψ—heading angle of the ship; sst—bow thruster reference value (−1…1); $AZ_{n},AZ_{\alpha }$—azipods’ thrust reference value (1…10) and orientation angle; $V_{w},\gamma_{w}$—velocity and angle of apparent wind.

Figure 9 shows the training ship trajectory and recorded histories of the key parameters of the port departure maneuver. The reference trajectory marked on the upper part of the figure by dashed line consisted of 4 waypoints. Their data are presented in Table 2. The longitude and latitude numbers are set in World Geodetic System (WGS) 84 format. One can observe satisfactory controller performance in the longitudinal and rotational channels, while, in the transversal channel, quality of the control is poor, especially in the final part of the maneuver. The control system working with very small levels of the set-point values for thrusters is extremely sensitive for wind gusts. This is important, particularly, for ships with high lateral area, like LNG carriers. Please note that the wind velocity measured by the anemometer should be scaled up to the size of ship model by square root of the scale multiplication factor ($\sqrt{24}$). Therefore, feedforward controller compensating wind influence seems to be necessary.

**Figure 10:**
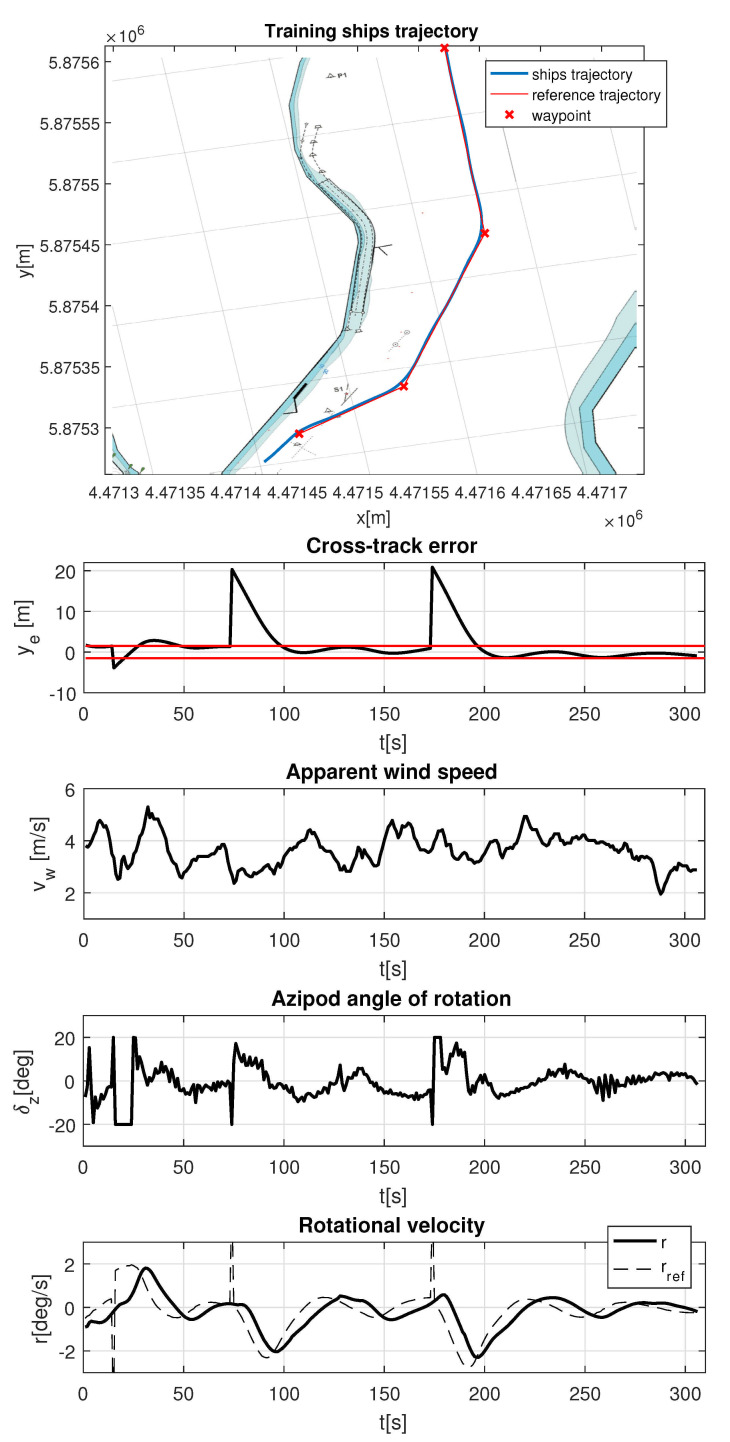
“Dorchester Lady” model MPC–LOS trajectory tracking. Upper part: Reference (red line) and measured (blue line) trajectories of the training ship, waypoints (WPTs) (marked by cross). Lower part: recorded signals marked with following symbols: $y_{e},v_{w},\delta_{z},r,r_{ref}$—cross-track error (“–” sign indicates that ship has reference trajectory on the port side), apparent wind speed, azipod angle of rotation (“–” sign indicates thet azipods are rotated to the port side), rotational velocity (“–” sign indicates counterclockwise rotation), and reference rotational velocity, respectively; reference rotational velocity is marked with dashed line.

Figure 10 shows MPC–LOS trajectory tracking results and recorded histories of the key parameters of the maneuver. Reference trajectory, marked on the upper part of the figure by red line, consists of four waypoints. They are presented in global Earth coordinate system. Their data are presented in Table 3. The longitude and latitude numbers are set in WGS 84 format. This way of the results presentation was used in order to emphasize good trajectory tracking performance. Cross track error (XTE) is then shown as a difference between ship’s and reference trajectory in meters. Admissible cross-track error, lower than ship’s breadth, is marked with the red line in the $y_{e}$ graph. It is shown that recorded cross-track error goes beyond the acceptable range only on turns, where XTE is not a parameter that can be used to assess the quality of regulation. The upper part of the Figure 10 shows that turns are made without overshoot and trajectory is tracked without oscillations. Apparent wind speed indicates the level of wind disturbance and is reflected in the azipods angle of rotation oscillations. Use of a feedforward controller in the future research is likely to minimize this effect. These oscillations are seen, because MPC controller is sensitive to the model and plant mismatch and the controller itself reacts to a fast-changing wind disturbance that has already occurred. In the lowest part of the figure, LOS rotational velocity reference (dashed line) and its tracking (solid line) are shown. A delay in the setpoint tracking close to the control horizon is observed.

The tests showed that one of the features of the LMI controller is the ultimate small size of the control matrix [3 × 6], and the results of the regulated speeds relative to the setpoints are within 20% overshoot. As the training vessel is to be a research vessel, it was decided that for high open water speeds a controller with MPC prediction would perform much better. Measured steady-state cross-track error is within predefined limit not exceeding ±ship’s breadth. The most important issue was to verify the results in terms of MASS, i.e., autonomy, safe harbor maneouvers, reference trajectory provided by a separate system tracking possibility, control process visualisation, and all the subsystems cooperation, rather than to develop and test in a detailed way an automatic ship control algorithms. This goal of the research has been achieved.

## 6. Discussion

The idea of MASS is a solution that will reduce the number of seafarers working on ships in the future and can even eliminate them. Today, Kongsberg Maritime augmented with Rolls-Royce Marine technology is leading the activities on a commercial level of autonomous shipping. Scientists all over the world are also looking for novel algorithms allowing for autonomous shipping. They have to face not only technical, physical, and control problems but also have to comply with existing legal requirements. Today, there is a lack of rules and guidance on how to build and operate autonomous vessels. The International Maritime Organization (IMO) is discussing this topic and adopted interim guidelines for autonomous MASS surface ships at its 101st session in 2019.

Currently, the legislation concerns the adoption of 4 degrees of autonomy (from the level where the ship’s systems support the decisions of the master present on the ship to level four defining an unmanned, self-manipulating and self executing ship). On IMO websites, the Commission informs about the planned regulation for 2020 on rights and rules appropriate to the new situation for research work and training conditions for MASS-type. An important aspiration of the new regulation may be the need to undertake training activities for both crews, operators, and vessel traffic control groups.

Conducted research, presented in this paper, allowed for creation of fully functional motion control system for MASS based on the training ship. This indicates the legitimacy of further research in the autonomous ship field. Development of algorithms for automatic control of the ship’s motion in various operating conditions is an essential shape of the process of seagoing autonomous merchant ship creation. MASS, except for favorable legal conditions, requires work on algorithms for determining a safe trajectory and their combination with a full-mission controller.

The challenge is, naturally, to build the reliable motion control system for autonomous full-size ship for her entire voyage from port to port. Promising results in this field have been achieved as shown in the draft in Reference [45].

In addition, one more aspect should be highlighted except the algorithms and calculation methods of control signals used. It seems to be even more important in the autonomous ship motion control technology. It is the measurement system. The experiments with the ATS described in this work have shown that gyrocompass, precise GPS in RTK mode, anemometer, and the reliable measurements of propulsors are sufficient to control scale model ship. In case of the full-size one, deliberate policy ought to be introduced to establish trustworthy measurement system for ship motion control.

## 7. Conclusions

The research presented in this work has shown that it is possible to control the motions of the autonomous ship in different stages of her voyage using LMI and MPC control paradigms. The experiments were conducted in the real environment using the scale model training ships. One of the more important aspect of the control system is to design trustworthy measurement structure to keep the autonomous ship aware of the environmental factors.

The most important issue was to verify the results in terms of MASS, i.e., autonomous rather than automatic ship control. The most important issue was to verify the results in terms of MASS, i.e., autonomous rather than automatic control of the vessel. Therefore, it is innovative to carry out a number of maneuvers on the lake to test autonomous vessel control considering the two methods LMI and MPC. This is the first step extremely necessary to test the algorithms based on the anti-collision solutions needed for autonomy according to MASS4. We plan to test other, more demanding control techniques and to assess which of the control algorithms will work best in autonomous ship control.

## Figures and Tables

**Table 1:** Training ship particulars.

Parameter	“Dorchester Lady”
Length overall L [m]	11.55
Breadth B [m]	1.80
Draft T [m]	0.50
Displacement D [T]	8.21
Max. speed u [kn]	4.1

**Table 2:** Departure maneuver waypoints (WPTs) data.

Waypoint No.	Longitude [m]	Latitude [m]	Heading [deg]
1	4,471,184.04	5,875,404.47	132.50
2	4,471,185.81	5,875,406.74	132.50
3	4,471,196.23	5,875,403.71	125.00
4	4,471,207.42	5,875,395.87	130.00

**Table 3:** Model Predictive Control (MPC)–Line-of-sight (LOS) trajectory tracking waypoints data.

Waypoint No.	Longitude [m]	Latitude [m]	Heading [deg]
1	4,471,452.27	5,875,295.22	047.0
2	4,471,537.91	5,875,334.05	065.0
3	4,471,604.28	5,875,459.39	025.0
4	4,471,571.31	5,875,611.15	350.0

## Data Availability

Data available on request due to restrictions eg privacy or ethical.

## Abbreviations

The following abbreviations are used in this manuscript:

AAWA	Advanced Autonomous Waterborne Applications Initiative
AIS	Automatic Identification System
ATS	Autonomous Training Ship
AVAL	Autonomous Vessel with an Air Look
COLREG	International Regulations for Preventing Collisions at Sea
GPS	Global Positioning System
DWT	Deadweight Tonnage
ECDIS	Electronic Chart Display and Information System
FRAM	Functional Resonance Analysis Method
GUI	Graphical User Interface
HMI	Human-Machine Interface
IMO	International Maritime Organization
LMI	Linear Matrix Inequalities
LMM	Last Minute Maneuver
LNG	Liquefied Natural Gas
LOS	Line-of-sight
MASS	Marine Autonomous Surface Ship
MPC	Model Predictive Control
NMEA	National Marine Electronics Association
PROMARE	Promoting Marine Research and Exploration
RTK	Real Time Kinematic
SLRT	Simulink Real-Time Toolbox
STPA	System-Theoretic Process Analysis
WGS	World Geodetic System
WPT	Waypoint
WSL	Water-Shore Line
XTE	Cross-track error
